# Supplementary material for: Platelet derived growth factor receptor β (PDGFRβ) is a host receptor for the human malaria parasite adhesin TRAP
Source: Sci Rep. 2021 May 31;11:11328. doi: 10.1038/s41598-021-90722-5 (PMC8166973; doi:10.1038/s41598-021-90722-5)
Supplement: Supplementary file 1 — Supplementary Information 1. [file 41598_2021_90722_MOESM1_ESM.pdf]

**Supplementary information for:**

# Platelet Derived Growth Factor Receptor β (PDGFRβ) is a Host Receptor for the human malaria parasite adhesin TRAP

Ryan W. J. Steel[1*&], Vladimir Vigdorovich[1*], Nicholas Dambrauskas[1], Brandon K. Wilder[1§], Silvia A. Arredondo[1], Debashree Goswami[1], Sudhir Kumar[1], Sara Carbonetti[1], Kristian E. Swearingen[2], Thao Nguyen[1], Will Betz[1], Nelly Camargo[1], Bridget S. Fisher[1], Jo Soden[3], Helen Thomas[3], Jim Freeth[3], Robert L. Moritz[2], D. Noah Sather[1, 4, 5, #], Stefan H.I. Kappe[1, 4,5, #]

[1] Seattle Children's Research Institute, Seattle, WA, USA
[2] Institute for Systems Biology, Seattle, WA, USA
[3] Retrogenix Ltd, Chinley, High Peak, SK23 6FJ, United Kingdom
[4] Department of Pediatrics, University of Washington, Seattle, WA, USA
[5] Department of Global Health, University of Washington, Seattle, WA, USA

* Equal contribution

[&] Present address: Infectious Disease and Immune Defence Division, The Walter and Eliza Hall Institute of Medical Research, Parkville 3052, VIC, Australia
[§] Present address: Vaccine and Gene Therapy Institute, Oregon Health & Science University, Beaverton, OR 97006

[#] To whom correspondence should be addressed:
Stefan Kappe stefan.kappe@seattlechildrens.org;
D.Noah Sather noah.sather@seattlechildrens.org

**Supplementary Figure 1: Characterization of recombinant *Plasmodium* TRAP ectodomains fragments.**

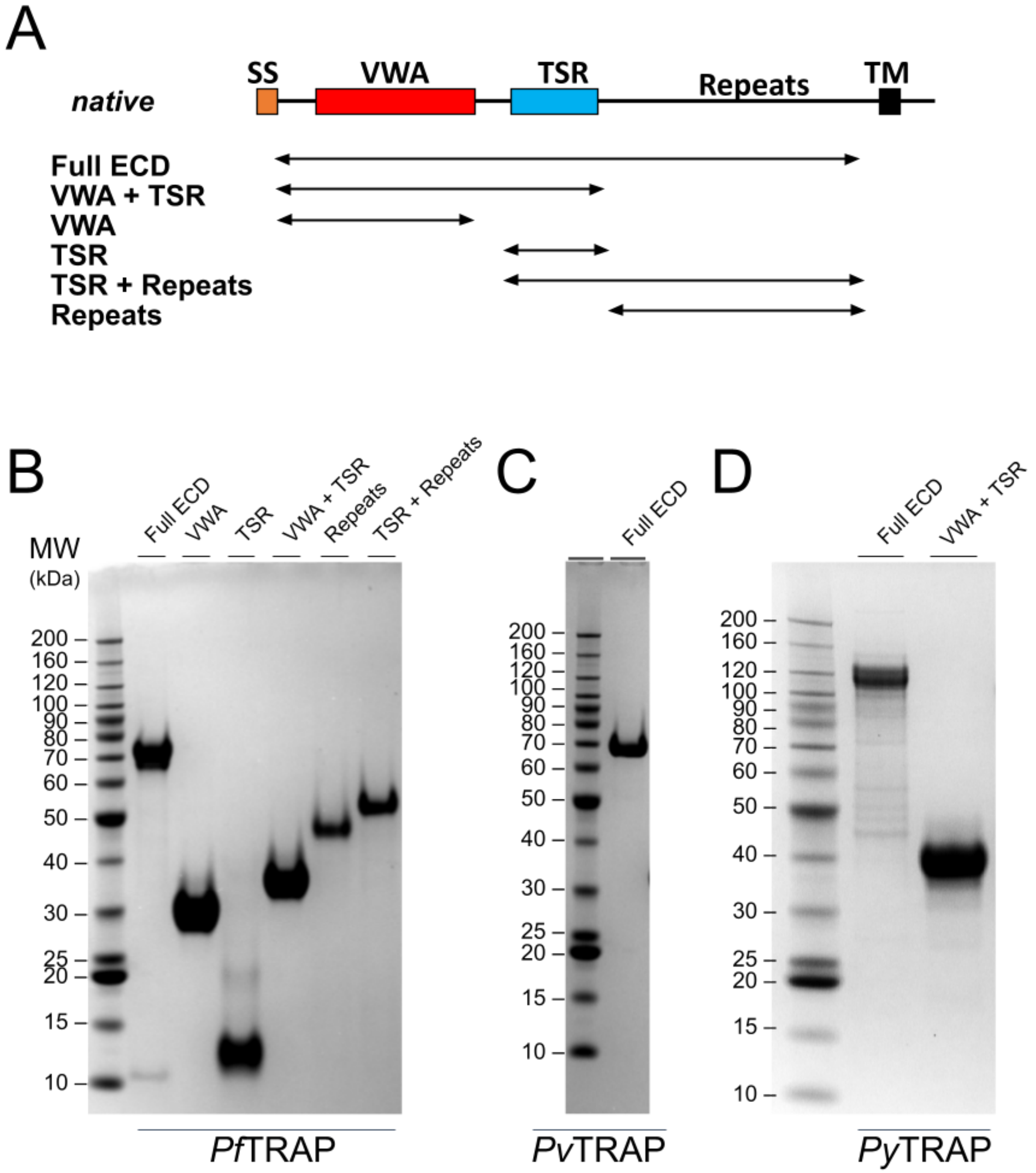


(A) Schematic representation of deletion constructs spanning the ectodomain of TRAP.

SDS-PAGE of the six constructs produced using the *P. falciparum* (B), *P. vivax* (C), and

*P. yoelii* (D) sequences. Labels for the molecular weight standards are shared across panels B–D.

**Supplementary Figure 2: PfTRAP binds to PDGFRβ expressed in HEK293 cells using the Retrogenix Cell Microarray platform.**

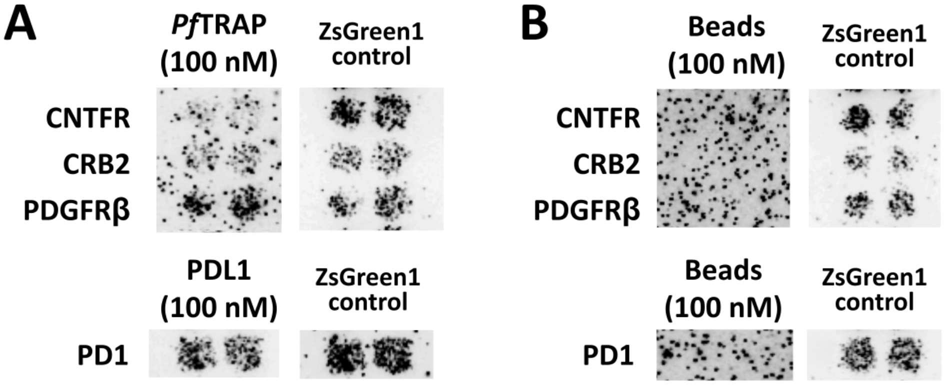


Hits from primary binding screens were re-arrayed for reverse transfection into HEK293 cells in duplicate for secondary specificity and reproducibility screens in conjunction with a ZsGreen1 reporter construct for successful transfection (right half of each panel). Fluorescent beads conjugated with PfTRAP full ECD identified weak, albeit specific binding to CNTFR- and CRB2-transfected cells, and strong binding to PDGFRβ-transfected cells (A) that was not observed with unconjugated beads (B). Positive control ligand, PDL1, oligomerized to fluorescent beads bound strongly and specifically to its known interaction partner PD1.

## Supplementary Figure 3: Analysis of ligand binding by flow cytometry.

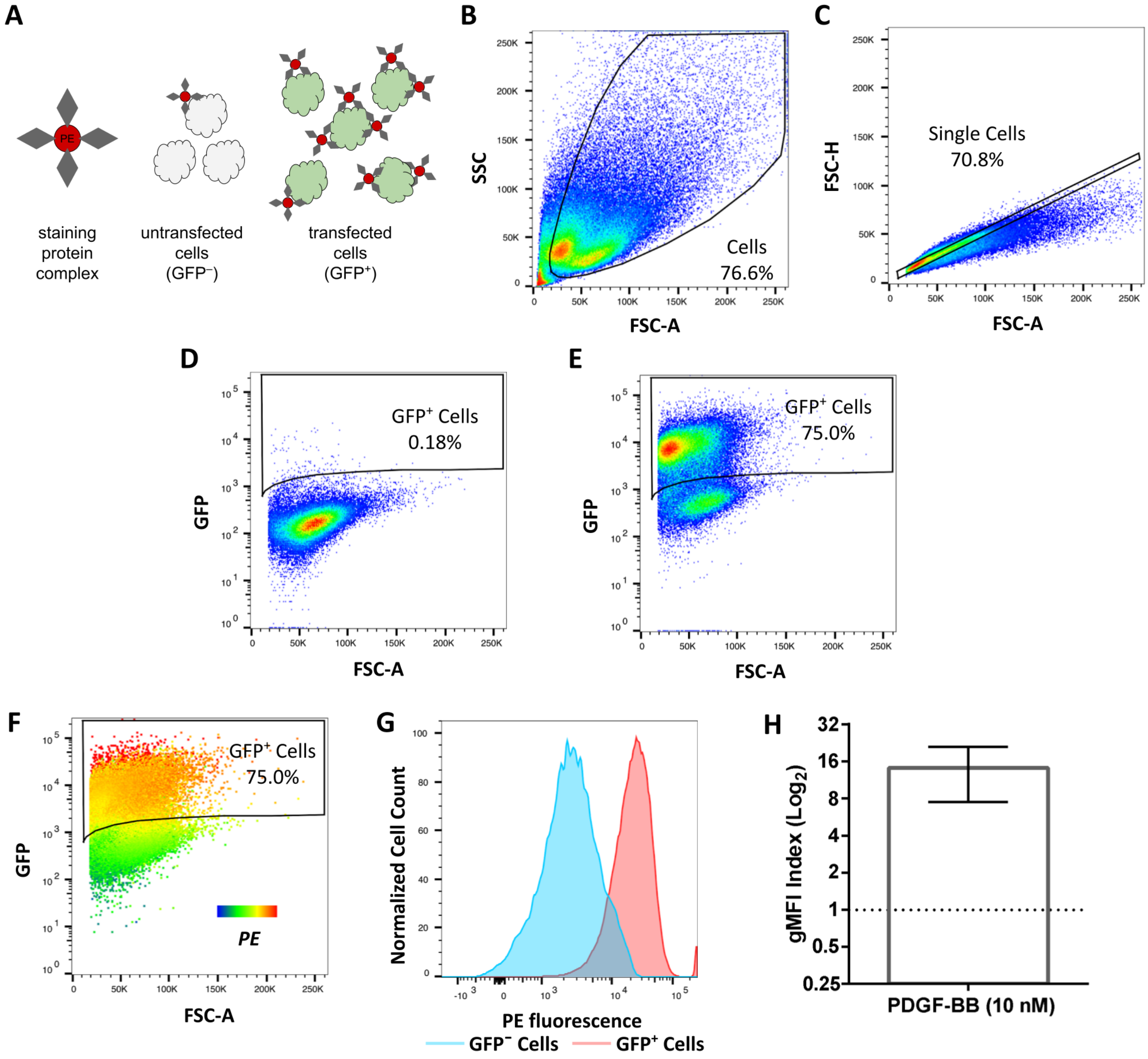


Biotinylated ligand (PDGF-BB in this example) was oligomerized around a streptavidin-conjugated phycoerythrin (PE) staining reagent (A, left) and incubated with HEK293F cells for 30 minutes before analysis by flow cytometry. This would produce a $GFP^+$ transfected population (A, right) expressing the PDGFRβ-GFP fusion and a residual untransfected $GFP^-$ population (A, middle). For analysis, cell-like events were first separated from debris (B) and single cells selected for analysis (C). A mock-transfected cell sample was used to set the $GFP^+$ transfected cell gate (D) from

which cells transfected with a GFP-fusion of PDGFRβ and untransfected $GFP^{-}$ cells from within the same sample were identified (E). The geometric mean fluorescent intensity (gMFI) of PE staining was used to determine the binding of the oligomerized staining reagent to the transfected and untransfected cell population. The positive control ligand (human PDGF-BB) bound strongly and preferentially to the PDGFRβ-transfected cell population (F, G). Specificity of binding to the transfected receptor population was measured as the ratio of the gMFIs from $GFP^{+}$ and $GFP^{-}$ cells, reported as the 'gMFI Index', and measurements were taken over multiple experiments to ensure the reproducibility of the results (F–H). Data in B–G show a representative experiment. Data in H arise from 7 independent experiments and represent the mean ± SD; the dashed line represents the gMFI index expected if no specific binding occurs (i.e., gMFI index = 1).

**Supplementary Figure 4: Titration of PfTRAP fragment binding to hPDGFRβ in the flow cytometry binding assay.**

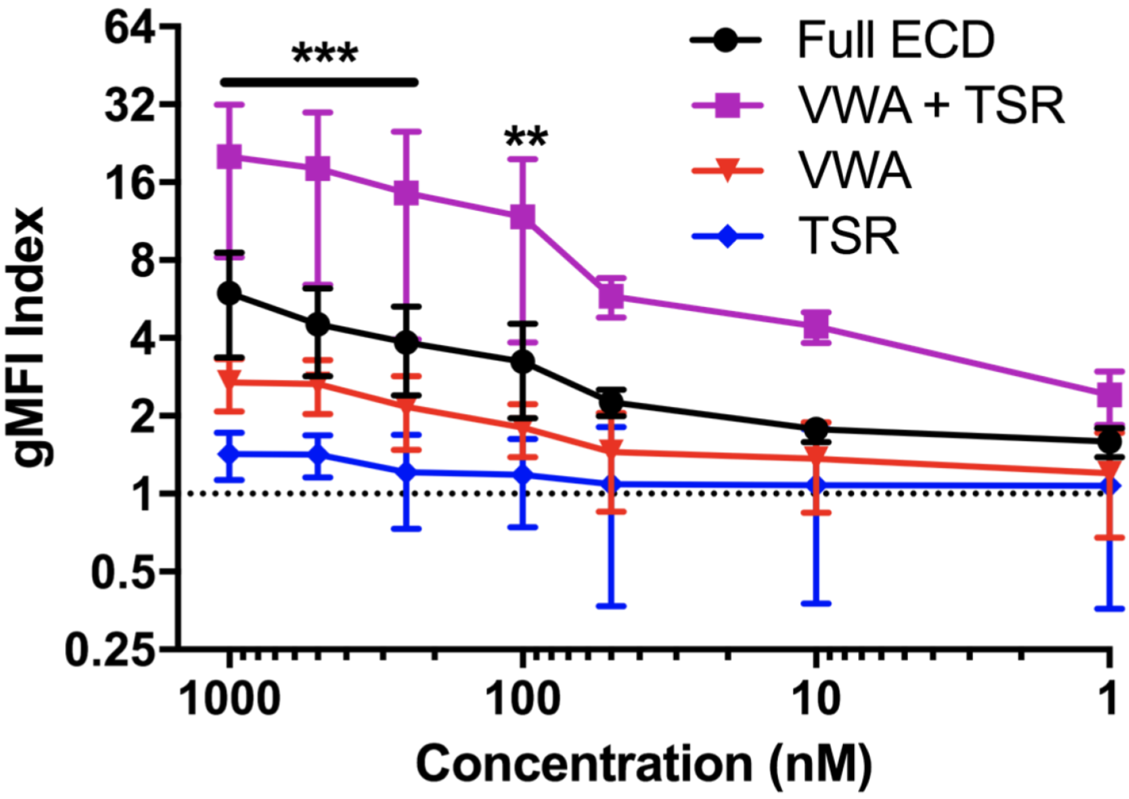


The gMFI index of VWA+TSR fragment bound to hPDGFRβ transfected cells was greater than all other fragments at concentrations at or above 100 nM. Data are the same as those in Figure 1 and represent the mean ± SD from at least five independent experiments; they are shown here in one plot to make the comparisons more convenient. Analysis by Two-Way ANOVA with Bonferroni's multiple comparisons test; asterisks indicate the VWA+TSR fragment is different from all others, **$p<0.01$, ***$p<0.001$.

**Supplementary Figure 5: Validation of anti-PDGFRβ mAb 2C5 and its use in *P. yoelii* passive transfer studies.**

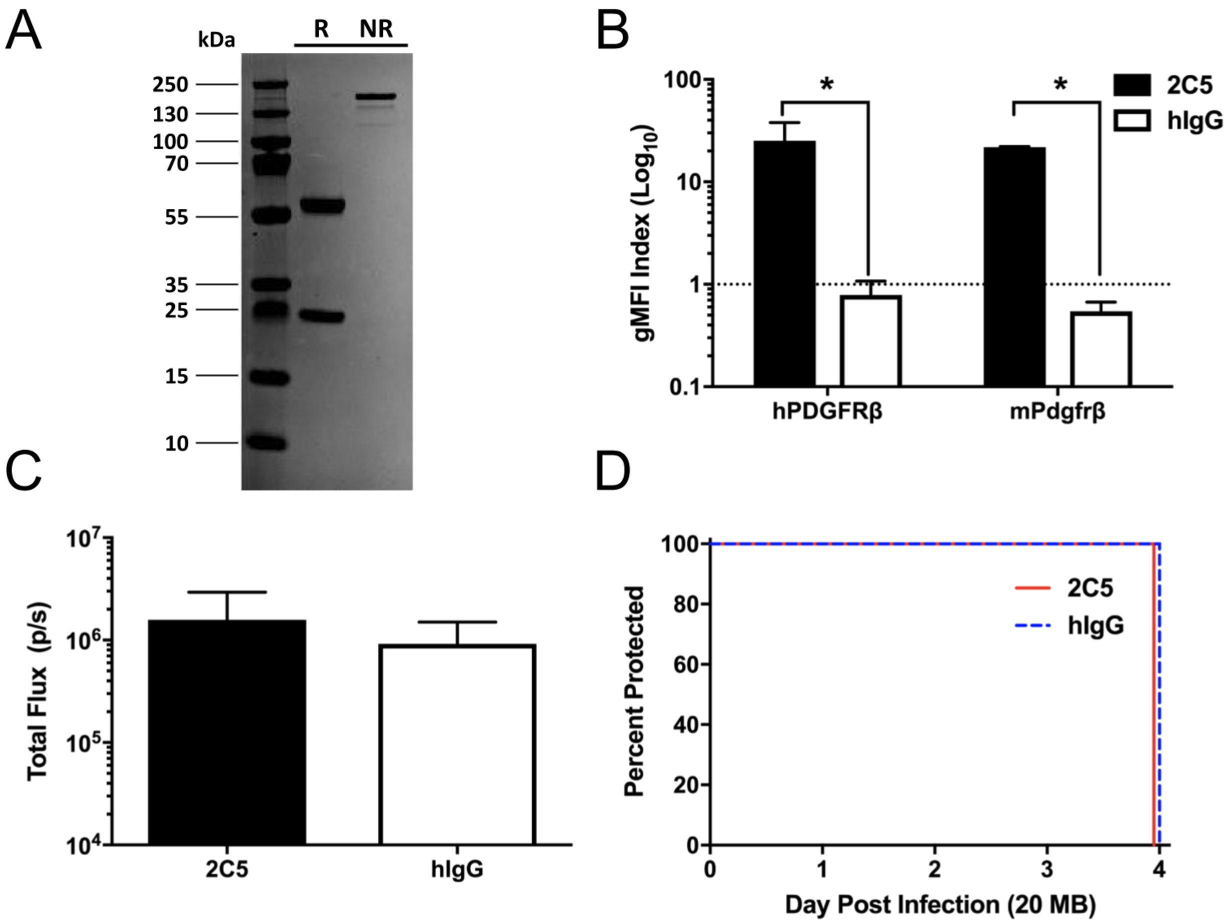


(A) mAb 2C5 was produced as a human IgG1 and analyzed by SDS-PAGE under reducing (R) and non-reducing (NR) conditions. (B) Cells were transfected with human or mouse PDGFRβ (hPDGFRβ and mPDGFRβ, respectively), then incubated with 100 μg/mL mAb 2C5 or isotype control before washing and staining with an anti-human antibody to measure mAb 2C5 specificity for PDGFRβ. Data are the mean ± SD from at least 2 independent experiments with analysis by t-test; *$p<0.05$. (C-D) Eighteen hours and one hour before challenge with 20 bites from *P. yoelii* infected mosquitoes, BALB/cJ mice received 800 μg mAb 2C5 (n=5) or isotype control (n=5; 1.6 mg of antibody total).

There was no difference in the malaria liver stage infection at 45 hours post infection nor any delay to blood stage patency.

**Supplementary Figure 6: Purified recombinant human PDGFRβ does not bind purified recombinant PfTRAP in biolayer interferometry experiments.**

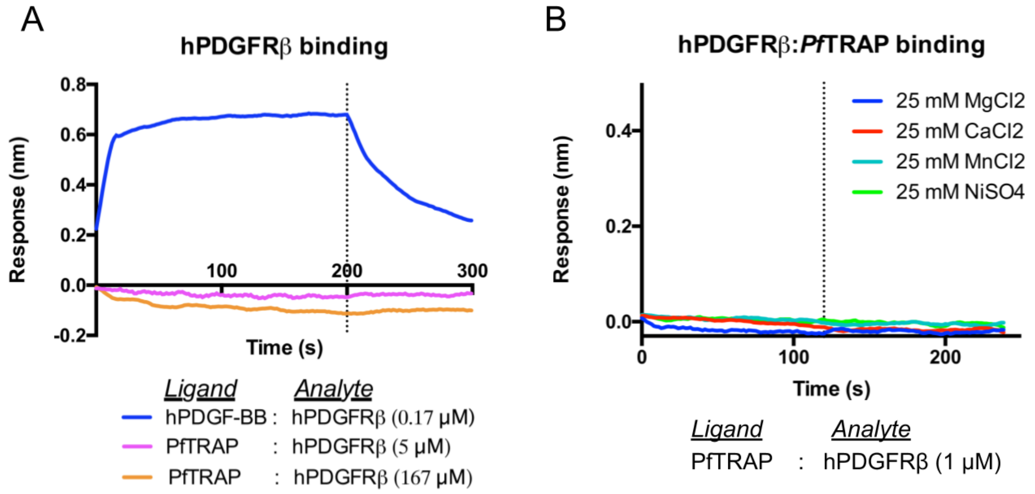


(A) Solutions containing recombinant biotinylated PfTRAP or hPDGF-BB (ligands) were used to derivatize streptavidin biosensors prior to incubating them in solutions containing recombinant hPDGFRβ (analyte) at the indicated concentrations for 200s, subsequently incubating them in reference buffer for another 100s. (B) Experiments using biotinylated PfTRAP as ligand were performed in the presence of 25-mM concentrations of divalent cations, as indicated in the legend. Sensorgrams in each experiment were reference-subtracted using data from an analyte-free reference well.

**Supplementary Figure 7: HEK293F cells express αv-integrin.**

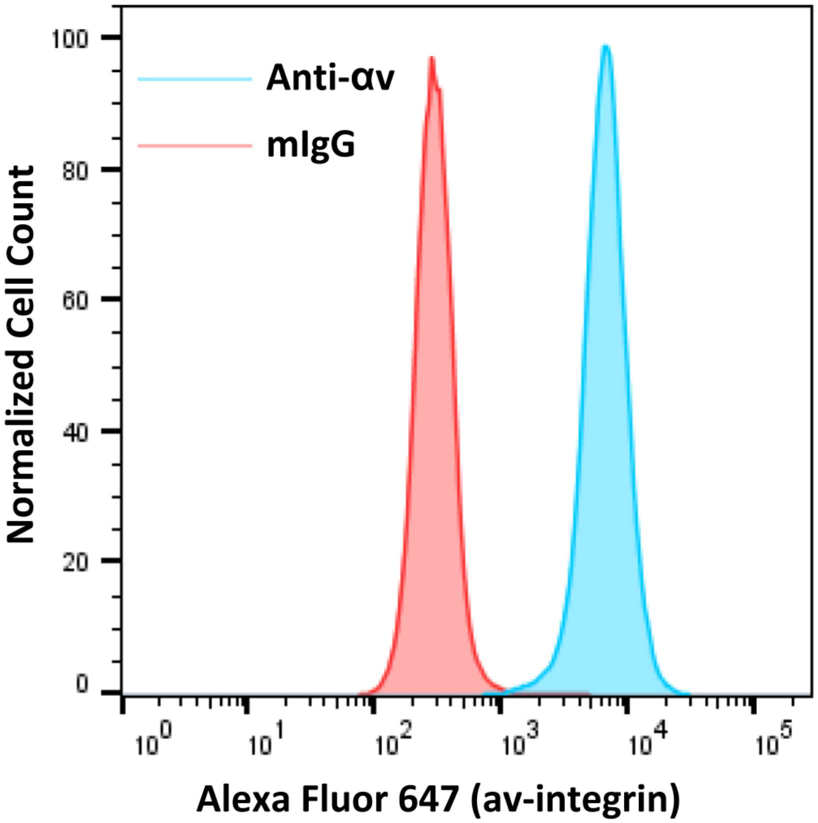


Untransfected HEK293F cells were incubated with 10 μg/mL anti-αv-integrin antibody or mouse IgG (mIgG) before washing and staining with an anti-mouse Alexa Fluor 647 antibody. Cells incubated with the anti-αv-integrin antibody were significantly brighter than the isotype control.

**Supplementary Table 1: Human Plasma Membrane proteins screened**

See the supplementary dataset file.
